# Evaluating the Healing Potential of J-Plasma Scalpel-Created Surgical Incisions in Porcine and Rat Models

**DOI:** 10.3390/biomedicines12020277

**Published:** 2024-01-25

**Authors:** Lilith Elmore, Nicholas J. Minissale, Lauren Israel, Zoe Katz, Jordan Safran, Adriana Barba, Luke Austin, Thomas P. Schaer, Theresa A. Freeman

**Affiliations:** 1 Department of Orthopaedic Research, Thomas Jefferson University, Philadelphia, PA 19107, USAjordan.safran@students.jefferson.edu (J.S.); 2 School of Osteopathic Medicine, Rowan University, Stratford, NJ 08084, USA; 3 Department of Clinical Studies, New Bolton Center, School of Veterinary Medicine, University of Pennsylvania, Kennett Square, PA 19348, USAtpschaer@vet.upenn.edu (T.P.S.); 4 Rothman Orthopaedic Institute, Philadelphia, PA 19107, USA

**Keywords:** cold atmospheric plasma, surgical site infection, periprosthetic joint infection, plasma scalpel

## Abstract

Cold atmospheric plasma devices generate reactive oxygen and nitrogen species that can be anti-microbial but also promote cell migration, differentiation, and tissue wound healing. This report investigates the healing of surgical incisions created using cold plasma generated by the J-Plasma scalpel (Precise Open handpiece, Apyx Medical, Inc.) compared to a steel scalpel in in vivo porcine and rat models. The J-Plasma scalpel is currently FDA approved for the delivery of helium plasma to cut, coagulate, and ablate soft tissue during surgical procedures. To our knowledge, this device has not been studied in creating surgical incisions but only during deeper dissection and hemostasis. External macroscopic and histologic grading by blinded reviewers revealed no significant difference in wound healing appearance or physiology in incisions created using the plasma scalpel as compared with a steel blade scalpel. Incisions created with the plasma scalpel also had superior hemostasis and a reduction in tissue and blood carryover. Scanning electron microscopy (SEM) and histology showed collagen fibril fusion occurred as the plasma scalpel incised through the tissue, contributing to a sealing effect. In addition, when bacteria were injected into the dermis before incision, the plasma scalpel disrupted the bacterial membrane as visualized in SEM images. External macroscopic and histologic grading by blinded reviewers revealed no significant difference in wound healing appearance or physiology. Based on these results, we propose additional studies to clinically evaluate the use of cold plasma in applications requiring hemostasis or when an increased likelihood of subdermal pathogen leakage could cause surgical site infection (i.e., sites with increased hair follicles).

## 1. Introduction

Many surgical procedures require a means to control or stop bleeding (hemostasis) into the surgical site. Hemostasis during surgery is commonly accomplished using mono- and bipolar electrocautery devices that generate intense heat to cauterize blood vessels. Unfortunately, the use of these devices is necessary but can impair healing, cause skin damage, produce charring, and result in tissue scarring [1]. In contrast, cold plasma technology has been explored as an alternative way to control bleeding through platelet activation and blood coagulation [2,3] without intense heating or extensive tissue damage. Cold plasma is a complex mixture of biologically active components, including charged particles, an electric current, UV radiation, and reactive oxygen and nitrogen species (ROS/RNS). The reactive species produced by cold plasma have also been shown to have bactericidal properties and enhance wound healing [4,5]. A cold plasma jet creates plasma using a high-powered electric current generator and helium gas to deliver the plasma to the tissue. By comparison, current traditional electrocautery devices, such as a Bovie device, use the same type of generator to deliver a high-energy radiofrequency (RF) spark directly to the tissue, causing localized heating and tissue damage [6].

Thus, this study was initiated to determine if the use of a cold plasma scalpel to create surgical incisions could achieve hemostasis without the side effects of electrocautery and undergo wound healing comparable to a steel scalpel. To our knowledge, cold plasma has not been studied in creating surgical incisions but only during deeper dissections. Cold plasma has been used to dissect osteoseptocutaneous flaps, fasciocutaneous flaps, and musculocutaneous flaps without any observed complications [7]. However, the optimal settings to create full-thickness skin incisions have not previously been defined or reported. Other biomedical applications of cold plasma have been investigated, including infection control, chronic wound healing, coagulation, deep dissection in plastic and vascular surgery, and eradication of malignant disease [8,9,10,11,12].

Cold plasma, with a range of plasma devices, has been employed in various medical fields, such as oncology, gastroenterology, dermatology, and plastic surgery. Of note, the term “cold plasma” is generically applied to a wide variety of devices that generate cold plasma using differing gas sources, including helium, oxygen, and air, or multiple settings for power or intensity and rates of gas flow. This results in the ability to fine tune cold plasma’s composition of ions and reactive species and intensity of energy for specific applications. Specifically, when cold plasma is generated at low power and is applied for short durations, wound healing and immune cell function can be stimulated, while at higher powers, bacterial infections or cancer cells can be eradicated [13,14,15]. It is generally accepted that the differential production of ROS/RNS accounts for the biological effects described above [16]. Other actions attributed to the cold plasma-generated reactive species when interacting with tissues include the modification of proteins, activation of molecular signaling pathways [17,18,19], cell proliferation, and tissue regeneration [20], which can also be stimulated by cold plasma. At higher power intensities or when treatment times are increased, DNA damage, lipid peroxidation [21], and cell death have also been reported [3,22,23].

Current methods of preoperative surface skin sterilization are inadequate to eradicate bacteria resident in dermal appendages (hair follicles and sweat and sebaceous glands) that lie beneath the skin surface. As the incision is created, pathogens from these structures can be released into the surgical site via scalpel transfer or through the flow of lymph wound fluid from the tissue [24,25]. The use of a cold plasma scalpel to create an incision has the potential to limit this, as the reactive species generated will make contact with the bacteria as it passes through the tissue. Thus, by creating surgical incisions with cold plasma, the antimicrobial nature of the reactive species generated can be exploited to enhance surgical site sterility [13,15].

Taken together, we propose that surgical incisions that limit the blood, wound fluid, and subdermal pathogens that flow into the surgical site could be beneficial to infection prevention, especially in areas with large numbers of hair follicles and sweat glands that harbor greater numbers of subdermal pathogens. However, compromising the wound healing process would abrogate these benefits. Therefore, as a first step, this study first determines the settings required to operate a cold plasma scalpel to create an incision smoothly with the least tissue damage. Next, as wound healing is of paramount importance, we compare wound healing to the gold standard of incisions created with a steel scalpel in porcine and rat models.

## 2. Materials and Methods

Ex vivo trials for surgical plasma setting optimization—We chose to employ the J-Plasma Precise Open (Apyx Medical, Clearwater, FL, USA) cold plasma scalpel, an FDA-approved helium plasma device used to coagulate and ablate soft tissue during open surgical procedures. To determine the optimal J-Plasma power (100% = 40 W) and gas flow (L/min) settings to create a full depth incision, an ex vivo model of skin was employed. Freshly harvested porcine or rat skin tissue samples from an unrelated study were acquired and kept on ice until the experimental procedure. Samples were placed on a silicone block over a ground plate connected to the J-Plasma device and moistened with 10 mM phosphate-buffered saline (PBS), pH 7. Incisions were created at variable gas flow and powers using the J-Plasma scalpel. At the end of the experiments, tissues were fixed in 4% paraformaldehyde > 24 h, dehydrated in graded ethanol (70 to 100%), and processed for either SEM or embedded in paraffin. Subsequently, 5 μm sections were acquired for histological analysis using Masson’s trichrome and hematoxylin and eosin (H&E) stains. Rat cadavers were also employed for the ex vivo testing of plasma settings. Rats were placed on the ground plate and 1.5-inch incisions were created at 30 or 40% (15 or 20 W) power at gas flows of 3 or 4 L/min.

Analysis of chemical species in plasma-treated liquid—To characterize the concentration of the reactive species present in J-Plasma, liquid saline was treated with J-Plasma, and its nitrite and hydrogen peroxide concentrations were determined. A solution of 3 mL of 0.9% sodium chloride was added to a flat-bottomed glass dish and surface treated with J-Plasma 10 mm away at either 30 or 40% power with gas flow settings of 3, 4, or 5 L/min for 3 min. The treated liquid was collected and prepared in a 96-well plate for the quantification of species. For the detection of nitrite species, 50 μL of Griess reagent (Sigma Aldrich, St. Louis, MO, USA) was added to 50 μL of J-Plasma-treated saline, incubated in the dark for 30 min, and measured at 548 nm on a Tecan Infinite M1000 spectrometer. For the detection of hydrogen peroxide, 100 μL of potassium iodide and 50 μL of 10 mM phosphate buffer at a pH of 7 was added to 50 μL of J-Plasma treated saline, incubated for 30 min, and measured at 390 nm. Values were converted to μM using a standard concentration curve.

Ex vivo tissue preparation for scanning electron microscopy (SEM) analysis—J-Plasma versus steel scalpel incisions were also assessed by SEM to visualize their effects on collagen fibrils and bacteria during incision creation. Incisions were created in excised skin from porcine or rat models, then fixed in 4% paraformaldehyde for at least 24 h before serial dehydration with a graded series of ethanol from 70 to 100%, with each phase lasting at least 3 h, then dried 48 h or longer. The samples were then sputter coated with platinum and imaged with a Tabletop Hitachi scanning electron microscope. The tissue surrounding the incision was then excised and prepared for SEM imaging as described above. SEM images were used to compare incisions.

Ex vivo rat bacteria injection incision trial—Fresh rat cadavers were injected with 300 µL saline containing 108 colony-forming units (CFUs)/mL of Staphylococcus aureus (ATCC 25923) into the dermal region of the skin. Incisions were created minutes after using either a steel or plasma scalpel, after which the surrounding tissue was immediately excised and fixed in 4% paraformaldehyde overnight. Tissues were then prepared for SEM. SEM images were used to visualize bacterial structural integrity in incisions created with J-Plasma versus a steel scalpel.

In vivo porcine trial for surgical plasma optimization—Following International Animal Care and Use Committee (IACUC) approval consistent with ARRIVE guidelines by the University of Pennsylvania, studies were performed in a porcine model at Penn Vet New Bolton Center Hospital for Large Animals. The porcine skin has remarkable similarities to human skin and is used widely in dermatological and wound healing studies [26]. For this comparative study, we used one castrated male 35 kg Yorkshire pig. The animal was kept in a 6 × 6 ft pen with straw bedding and visual, olfactory, and auditory contact to herd mates in adjacent pens. The pig was premedicated intramuscularly with 0.2 mg/kg butorphanol (Turbogesic, Zoetis, Parsippany, NJ, USA), 0.3 mg/kg midazolam (Midazolam, West-Ward, Eatontown, NJ, USA) and 0.015 mg/kg dexmedetomidine (Dexdomitor, Zoetis, Parsippany, NJ, USA) mixed in one syringe. After onset of appropriate sedation approximately 15 min after drug administration, an intravenous catheter was placed into the auricular vein and 2 mg/kg ketamine (Ketathesia, Henry Schein, Melville, NY, USA) was administered intravenously. Anesthesia was maintained with isoflurane (Isothesia, Henry Schein) in oxygen. Intravenous crystalloid solution (Normosol, Abbott Laboratories, Abbott Park, IL, USA) was administered at a rate of 3 mL/kg/h. The surgical site was shaved and prepped for aseptic surgery. Plasma full-thickness incisions were made along the animal’s dorsal flank with the J-Plasma device at 40% power, gas flow 3 L/min. Plasma, bipolar electrocautery, and steel scalpel (#11) full-thickness incisions were made 10 cm apart. Incisions were closed using #3 polyglactin 910 (Vicryl, Johnson and Johnson, Bridgewater, NJ, USA) in a simple continuous pattern for the subcutaneous tissue layer followed by #2 poliglecaprone 25 (Monocryl, Johnson and Johnson, Bridgewater, NJ, USA) in an interrupted pattern and dressed with a sterile dressing. The animal received buprenorphine (0.01 mg/kg IV or IM) pre- and post-operatively (SID-QID); fentanyl (2.5 mcg/kg/h transdermal), which was removed after 72 h, and flunixin meglumine (1.1 mg/kg IV or IM) SID for 3 days beginning the day of surgery for pain management. Wound healing was documented using photography, and aseptic full-thickness skin biopsy samples from each incision were taken at post-operative day 19 or 33 under general anesthesia. Biopsies from both time points were fixed >24 h in 4% paraformaldehyde (Sigma, St. Louis, MO, USA).

In vivo rat wound healing trial—Sixteen male Wistar rats weighing about 400 g were housed under standard conditions following IACUC guidelines with the approval of the Thomas Jefferson University IACUC committee. Each rat acted as its own control, comparing the standard-of-care steel scalpel incision on one side of the dorsum with the J-Plasma Precise Open instrument (Apyx Medical, Clearwater, FL, USA) incision on the opposite side. After anesthesia with isoflurane, animals were administered a 1 mg/kg dose of slow-release buprenorphine. Rat dorsa were shaved with electric clippers, and the skin was prepped with iodine and allowed to dry prior to marking 2 cm lines on the skin with a surgical marker on the lateral dorsum. On the left dorsum, an 11-blade steel scalpel was used to make a full-thickness incision, and on the right dorsum, the J-Plasma with a power level of 40% and gas flow of 3 L/min was used to make a second incision. Full-thickness incisions were made in dermal layers only, with their depth not penetrating beyond the myofascial tissue. Both wounds were sutured with non-absorbable 5-0 ethicon (Med-Vet International, Mettawa, IL, USA). Animals were monitored daily post-operatively, and photographs of the external appearance of the wounds were taken daily to monitor the progression of wound healing for each incision. If the rat chewed on the wound or otherwise bothered with it, that animal was excluded. Animals were euthanized with CO_2_ at days 3, 8, or 20 post-operatively (n ≥ 4 rats for each time point), and the tissue around the incision was excised and fixed >24 h in 4% paraformaldehyde (Sigma, St. Louis, MO, USA).

Grading of external healing of incisions in rat model—The images acquired daily of incisions to monitor external wound healing were provided to 4 blinded graders. The Southampton wound grading system [27] was modified to adjust their alphabetical descriptions, which were assigned a numerical value for quantitation purposes (Table 1). This system was used to grade images of both scalpel and plasma wounds from days 3 and 8 post-incision. Images from post-incision day 20 scalpel and plasma incisions were also given to the reviewers, and the Stony Brook Scar Evaluation Scale was used to grade these images [28].

Histology—After fixation with 4% paraformaldehyde for 24 h, samples underwent paraffin infiltration using the Tissue-Tek VIP processor. The orientation of the sample when embedding in paraffin was bisected perpendicular to, and in the center of, each incision, then embedded with the bisected side down into a paraffin wax block for sectioning (Thermo Fisher Scientific Inc., Waltman, MA, USA). Serial 5 μm transverse sections originating from the center of the incision were stained with toluidine blue, H&E, or trichrome stains (Thermo Fisher Scientific Inc., Waltman, MA, USA). Slides were then mounted with Permount (Thermo Fisher Scientific Inc., Waltman, MA, USA).

Image Analysis—Microscopic images were acquired using a Motic BA300Pol light microscope and the Jenoptik M50 ProgRes camera (Jupitor, FL, USA) or Nikon Eclipse (Nikon, Inc., Melville, NY, USA) with the Q-imaging Eclipse (Q-imaging, BC, Canada) at 4× and 10× magnification as 24-bit TIFF files. To objectively assess and quantitate wound healing, automated image analysis was performed at days 8 (correlating to the end of proliferative phase of wound healing) and 20 (end-stage wound healing) post-incision. Images of trichrome- and toluidine blue-stained sections were imported to Image-Pro Plus Ver. 7 (Media Cybernetics, Silver Spring, MD, USA) for analysis. As outlined by Sulthana et al. and others [29,30], to assess healing status, analyses of histological parameters should include quantifications of the amount of granulation tissue, immature vs. mature collagen, cellularity, and immune infiltrate. Instead of giving arbitrary qualitative values for minimal, moderate, and profound amounts, we used a quantitative assessment by the measurement of 3–4 images at multiple random locations along each incision for each type of measurement from at least 3 different animals/timepoint/treatment. Most analyses were paired such that incisions from both devices were performed on each rat dorsum.

As healing was not uniform along the 2 cm incisions, several sections (2–3 slides) were taken at multiple locations (4 or 5) along the incision length at 200–300-micron intervals from the center in both directions. The width of granulation tissue was measured on sections from areas that had complete re-epithelialization. Using the manual draw tool to define the edges of the granulation tissue by their color of staining (light blue compared to the darker blue normal dermal tissue), the average, minimum, and maximum distance between two lines was then calculated automatically by the Image Pro Plus software Ver. 7 (Media Cybernetics, Silver Spring, MD, USA) to generate the average width (as illustrated by red arrows) of the granulation tissue. For granulation tissue width, collagen maturity was assessed by extracting the green color channel from RGB trichrome images of day 8 wounds, yielding a grayscale image. A standardized rectangular region of interest (ROI) was created for all images around the wound site. Set thresholds were applied to all images to assess the areas comprising either the darker mature collagen or the lighter regions of immature collagen. Cellular infiltrate within granulation tissue was calculated using the toluidine blue-stained sections and a custom macro in ImagePro to count blue cells based on intensity and size filters. Further analysis of toluidine blue histology was conducted to count mast cells within the granulation tissue, with parameters set to account for the larger cell size and darker purple/pink intensity of mast cells. Conditions for each macro were determined by testing for accuracy on multiple images before applying it to all images in the dataset.

Statistical Analysis—All data were entered into the GraphPad Prism 8 (GraphPad Software, San Diego, CA, USA) statistical software. Images from the blinded grading of external wounds or histology sections were assessed by two-tailed *t*-tests; when images were from the same animal, the test was paired. Data were tested for normality using the Shapiro–Wilk normality test (alpha = 0.05, and a *p*-value of <0.05 was determined as statistical significance. For the blinded review of images, a Chi-square test was used.

## 3. Results

### 3.1. Determining the Optimal Settings for the J-Plasma Scalpel

**Figure 1:**
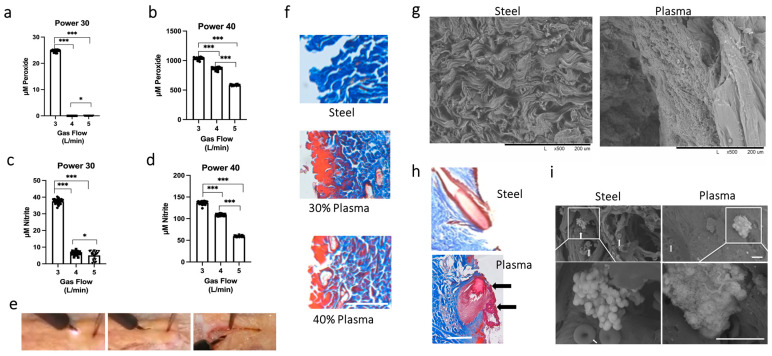
Comparison of reactive species and incision characteristics of steel versus J-Plasma scalpels. J-Plasma at gas flows of 3, 4, or 5 L/min was tested for the presence of peroxide and nitrite at 30% (**a**,**c**) or 40% power (**b**,**d**), respectively. (* = *p* < 0.05, *** = *p* < 0.001) (**e**) Series of images taken as plasma scalpel creates incision through porcine skin. The first picture taken as plasma ignites at initiation, followed by gliding through tissue, with the last image being the resultant incision with a smooth cut. (**f**) Comparison of reactive species penetration by trichrome staining of tissue cut with steel or plasma scalpels at 30% or 40% power. (**g**) Scanning electron microscopy (SEM) comparing porcine tissue cut with steel or plasma scalpels. (**h**) Trichrome stain of incision cut with steel or plasma scalpels at 40% power exhibits “sealing” of hair follicle by collagen fibril fusion (black arrows). (**i**) SEM of rat dermal tissue incision after injection of S. aureus at low magnification shows individual collagen fibril strands in steel scalpel incision vs. fused smoothed surface after plasma incision. Individual red blood cells (white arrows) are also more numerous in steel scalpel incision. White boxes indicate small colonies of bacteria shown at higher magnification in the lower panel, whereas a deterioration of the bacterial colony surface is evident after plasma treatment (mag. bar on histology sections = 1 mm).

As the interaction of cold plasma depends on tissue type, we determined it was paramount to establish cold plasma power settings that created a smooth incision through the skin. Secondly, we hypothesized that producing the greatest amount of ROS/RNS for the incision would enhance the likelihood that it would have microbicidal capabilities. Thus, various combinations of power and gas flow settings on the J-Plasma device were tested. It was determined the scalpel could not smoothly incise the tissue at powers less than 30%, and powers higher than 40% caused tissue charring. Similarly, gas flows of less than 2 L/min did not create a satisfactory incision. Therefore, we proceeded with only 30 or 40% power to determine ROS/RNS generation (nitrite and peroxide). Concentrations were measured in a 0.9% saline solution after treatment with the J-Plasma device at either 30 or 40% power at gas flows of 3, 4, or 5 L/min. We found that 30% power at a gas flow of 3 L/min generated the highest average concentration of H_2_O_2_ (24 ± 0.09 μM) and nitrite (37.49 ± 0.32 μM) (Figure 1a,c). No detectable peroxide species and low nitrite species were observed at gas flows of 4 and 5 L/min. A gas flow of 3 L/min also generated the highest peroxide (1033 ± 4.38 μM) and nitrite (136.3 ± 0.81 μM) concentrations at 40% power. Gas flows of 4 and 5 L/min yielded significantly lower concentrations of these species (Figure 1b,d). Overall, the highest amounts of peroxide and nitrite were generated at 40% power with a gas flow of 3 L/min. An image series of the plasma scalpel creating an incision is shown in Figure 1e.

To determine the effect on tissues after incision, ex vivo tissue samples from porcine or rat models were treated with a steel scalpel or a J-Plasma scalpel at 30 or 40% power with a gas flow of 3 L/min (Figure 1f). Trichrome staining of the incision made by the steel scalpel shows blue-stained, wavy collagen fibrils characteristic of the loose connective tissue of the dermis (top image). In contrast, tissues cut with the J-Plasma scalpel have areas of red staining along the incision (left side). We previously described this effect of plasma-generated reactive species modifying the affinity of the acid dyes in the trichrome stain [16]. Briefly, the basic nature of collagen is altered, causing a change in the affinity of the dyes such that the blue staining is replaced by red. Thus, the red color represents a rough estimate of reactive species penetration into the tissue. Histology showed that the depth of red staining (penetration of the species) was greater at 30% power compared to 40% power. This could have occurred because the scalpel moves slower through the tissue at 30% power, and therefore, the tissue is exposed to the plasma for a longer time, as it had been noted that making incisions at 40% power was much smoother and quicker than at 30%. Another observation was that the red-stained collagen fibrils also seemed fused together when compared to the blue-stained individual thread-like fibrils of collagen observed in the image of the tissue created with the steel blade scalpel.

### 3.2. Scanning Electron Microscope Results Support Plasma Fusion of Collagen Fibrils and Disruption of Bacterial Structure

To further investigate the fusion effect, scanning electron microscopy (SEM) of the cut collagen fibrils along the incisions was performed. Incisions created with steel or the plasma scalpel at the optimal setting of 40% power at 3 L/min were analyzed (Figure 1g). The SEM images clearly show the loose cut ends of the collagen fibrils after being incised with the steel scalpel. Conversely, after being incised by the plasma scalpel, the collagen fibrils have a smooth sheet-like appearance, confirming fibril fusion. Figure 1h shows the additional histology of a hair follicle in the plane of the incision with each scalpel type. Once again, the incision created with the plasma scalpel has the appearance of creating a fusion or sealing of the follicle (arrows) when compared to the incision created with the steel scalpel.

To assess what would happen to bacteria that come into contact with the plasma scalpel during the process of making the incision, we injected live S. aureus bacteria into the rat dermis before creating an incision. Within minutes after injection, an incision was made through the injected tissue with either the steel or plasma scalpel, after which the whole area was incised and prepared for SEM. SEM images of the incision made with the steel scalpel again clearly show the wavy individual fibrous “threads” of collagen. In contrast, as observed by histology, the collagen fibrils from incisions made with the plasma scalpel exhibited a smooth, sheet-like wall without any loose fibrils and with fusion of the collagen fibril ends. This smooth, fused topography, in addition to the decreased number of red blood cells (white arrows), indicates both tissue sealing, which may prevent lymph seepage into the surgical site, and the hemostasis of small vessels. Closer inspection at higher magnification to identify the effect on the injected bacteria showed bacterial clumps along the sealed collagen fibril interfaces (white boxes). These bacterial colonies were unaffected by the steel scalpel, whereas bacteria in the incision made by the J-Plasma scalpel showed a marked deterioration of surface topology (Figure 1i).

### 3.3. In Vivo Incisions in a Porcine Model Comparing Electrocautery and J-Plasma to a Steel Scalpel

**Figure 2:**
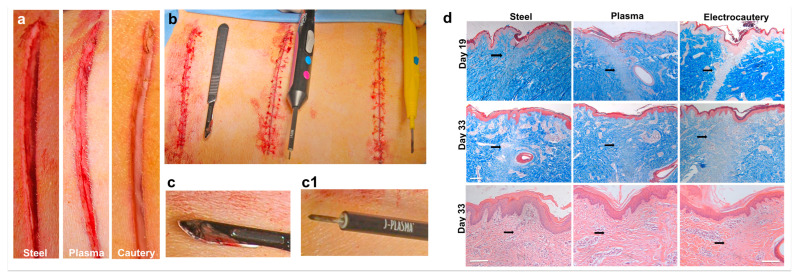
Comparison of incisions by steel, plasma, or electrocautery scalpels on porcine dorsa. External visualization of incisions immediately after incising with standard steel scalpel, J-Plasma, or electrocautery before (**a**) and after suturing (**b**) showing blood and fluid carry over on steel (**c**) versus plasma (**c1**) scalpels. (**d**) H&E and trichrome-stained histology images highlight differences in wound healing via granulation tissue appearance (black arrows) between wound healing at day 19 and day 33 post-incision with each device. 10× magnification (mag. bar = 1 mm).

To test the ability of the J-Plasma scalpel to create a hemostatic incision, an in vivo porcine model was used. One animal was used as a proof of concept to assess hemostasis and the gross healing of incisions created with the J-Plasma scalpel compared to a steel scalpel and electrocautery device. An image of each incision type is shown immediately after making the incision (Figure 2a) and after being closed with sutures, with each device being shown next to their respective incisions (Figure 2b). As expected, the scalpel incision resulted in the most amount of blood upon incision. In comparison, the incision with electrocautery resulted in almost no blood around the incision, and the incision made by the J-Plasma scalpel produced only minimal traces of blood. Additionally, blood remained on the steel scalpel post-incision creation (Figure 2c), in contrast to the J-Plasma scalpel (Figure 2c1), where blood was not visible.

### 3.4. J-Plasma Scalpel Showed Improved Tissue Healing Compared to Electrocautery in the Porcine Model

A histological analysis of the wound healing of the incisions created in the porcine model was performed on tissue sections from the incisions obtained on day 19 (biopsies) and day 33 after sacrifice using trichrome and H&E staining (Figure 2d; black arrows indicate granulation tissue, which stains light blue or white) in tissue incised by the electrocautery device. Conversely, the granulation tissue formation on sections treated by the steel scalpel or plasma scalpel was similar. After post-operative day 33, the incisional wounds created by the plasma or steel scalpels presented with well-arranged collagen. Overall, wound healing from the steel scalpel and J-Plasma incisions were similar in appearance and clearly more advanced than the tissue incised using electrocautery. At higher magnifications, H&E-stained sections from day 33 clearly show the newly remodeled collagen in the granulation area of the steel and plasma scalpel-incised tissue and the absence of this collagen in the tissue subjected to electrocautery (black arrows).

### 3.5. Plasma Scalpel Exhibits No Differences in External Wound Appearance When Compared to Steel Scalpel in the Rat Model

**Figure 3:**
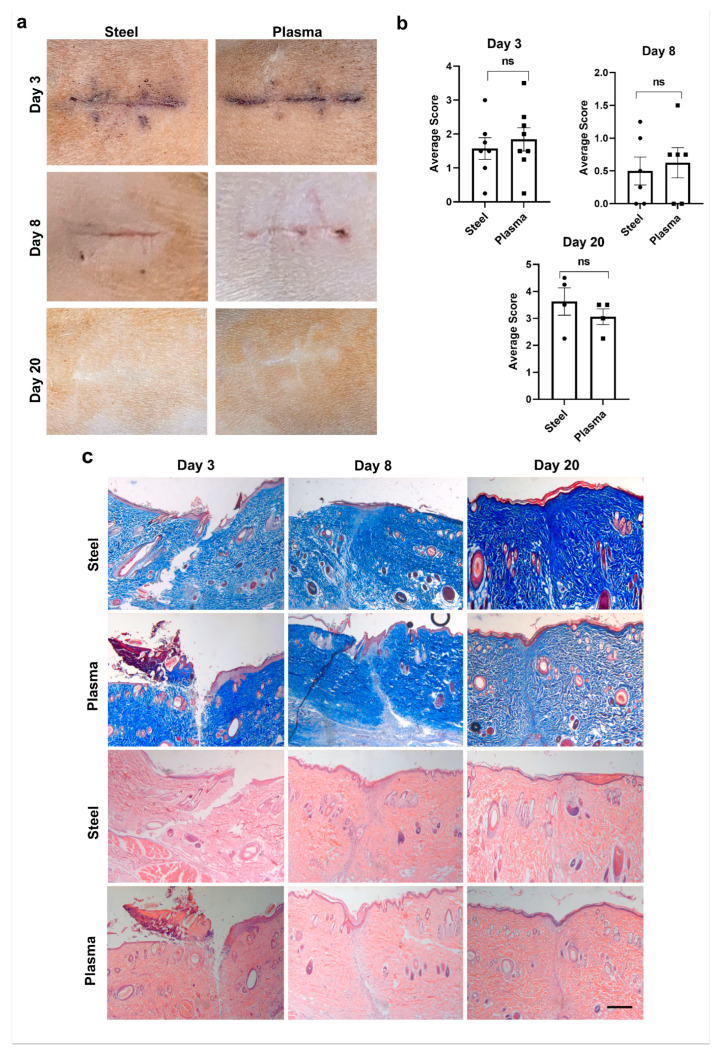
External wound grading and histological evidence of wound healing between incisions in an in vivo rat model made with steel or J-Plasma scalpels. (**a**) Representative images of days 3, 8, and 20 depicting external wounds caused by steel or plasma scalpels cutting 2 cm incisions. (**b**) Graphs showing the results from blinded wound grading using the Southampton Scoring System for day 3 (*p* = 0.44), day 8 (*p* = 0.70), and the Stony Brook Scar Evaluation Scale for day 20 (*p* = 0.21). (**c**) Trichrome- (upper 2 panels) and H&E- (lower 2 panels) stained sections give representative comparisons of wound healing at 3-, 8-, and 20-days post-incision with steel or J-Plasma scalpels (mag. bar = 1 mm; ns = not significant).

A more detailed analysis of wound healing was employed to test the effectiveness of the plasma scalpel after incision-making in a larger rat study. If future studies are to be planned and carried out in human subjects, cosmesis will likely be an important factor for both the surgeons and study participants. For this reason, a modified Southampton wound scoring system was employed for both day 3 and day 8 wounds. Representative images of external wound appearance are shown in Figure 3a at days 3, 8, and 20 post-incisions. Wound scores from four blinded graders resulted in no significant difference for day 3 (*p* = 0.44), with an average score for the steel scalpel of 1.5 ± 0.32 and 1.8 ± 0.34 for the plasma scalpel. On day 8, the steel scalpel scores averaged 0.50 ± 0.21, and plasma scores averaged 0.62 ± 0.23, and again scores were not significantly different (*p* = 0.70) (Figure 3b). On day 20, the average wound grades were 3.63 ± 0.50 for the steel scalpel and 3.06 ± 0.29 for the plasma scalpel. The results from day 20 also had no significant differences between the scars’ appearances (*p* = 0.21) as graded by the Stony Brook Scar Evaluation Scale. Overall, there was no significant difference between the external appearance of wounds and scars generated after incision with either the J-Plasma scalpel or the steel scalpel.

Histology on sections from wounds 3, 8, or 20 days after incision was used to visualize the stages of wound healing (Figure 3c) using trichrome (top 2 panels) and H&E (bottom 2 panels) staining. This was used to quantify the external appearance and wound healing in another re-producible manner. The progression of wound healing comparing the steel scalpel to the plasma scalpel showed little or no variation between the two modalities at any stage, including early healing (day 3), granulation (day 8), and the fully healed stage (day 20).

### 3.6. Quantitation of Histological Wound Healing Showed No Significant Differences between Steel and J-Plasma Scalpels in the Rat Model

**Figure 4:**
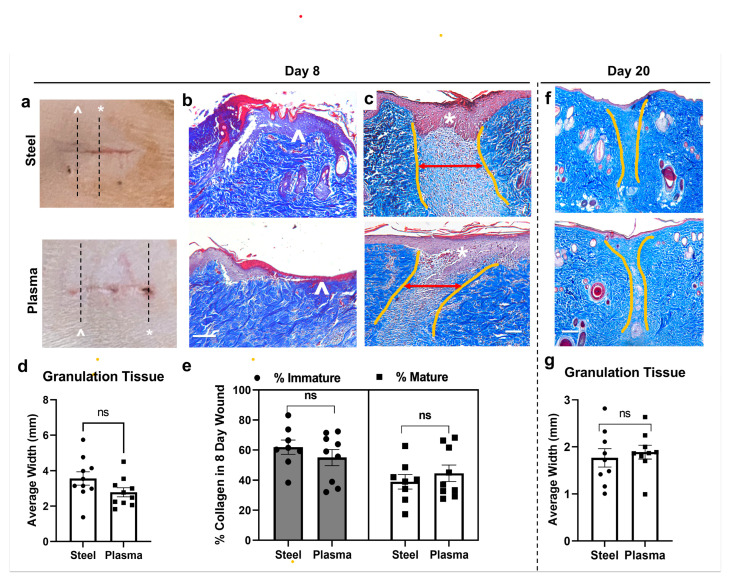
Comparison of granulation tissue amounts and composition in wound healing after steel or plasma scalpel incision. (**a**) Images of external wounds show areas with more (^) or less (*) healing along incision sites (2 cm). (**b**) Trichrome stain of wounds from steel or plasma scalpels on day 8 show differences in re-epithelialization corresponding to * and ^. (**c**) Lighter blue granulation tissue outlined by yellow lines were measured for average width (red two-sided arrows). (**d**) No significant (ns) difference was observed in a pairwise comparison of images (3–4 images/rat; *p* = 0.86; n = 3). (**e**) Analysis of trichrome-stained images for darker blue, mature collagen (peri-wound) or immature, light blue collagen in granulation area confirms no significant difference (*p* = 0.22). The same paired analysis of granulation tissue images on day 20 post-incision (**f**) also resulted in no significant difference (**g**) (*p* = 0.82, all mag. bars = 1 mm).

We quantified and compared the progression of the wound healing of wounds made by either a steel scalpel or J-Plasma using trichrome-stained sections on days 8 and 20 and analyzed the width of granulation tissue using ImagePro Plus (Figure 4a). Day 3 was excluded due to the lack of re-epithelialization in both steel and plasma scalpel wounds. As is evident from the external wounds’ appearance (Figure 4a), on day 8, incisions made with either scalpel type had areas with greater (white ^) or less (*) healing. Histology using trichrome staining shows a section from corresponding areas of worse (Figure 4b; white *, lack of epithelialization) and better (Figure 4c; white ^, thickened area of new epithelialization) areas of re-epithelialization along incisions from both steel and plasma scalpels made on the same rat. The steel scalpel wounds had an average width of 3.57 ± 0.35 mm, and a slightly smaller width was measured for wounds made with J-Plasma (2.78 ± 0.3 mm, *p* = 0.054; Figure 4d). Each dot represents a tissue section analyzed (2–3/rat, paired). Additional quantification of day 8 wounds to compare the ratio of mature to immature collagen was conducted on ImagePro Plus (Figure 4c). As a measure of tissue remodeling, our image analysis of mature collagen and immature fibrils (Figure 4e) showed that day 8 scalpel incisions contained 38.9 ± 4.82% mature collagen compared to plasma incisions with 44.6 ± 5.48% mature collagen, which also resulted in no significant difference between treatment groups (*p* = 0.22). At day 20 post-incision, granulation tissue from the wound bed in both incision types looked very similar (Figure 4f), and measurements confirmed this, with average granulation tissue width measuring 1.77 ± 0.62 mm for the steel scalpel and 1.89 ± 0.47 mm for J-Plasma (Figure 4g; *p* = 0.82).

### 3.7. Total Cell Infiltrate in Granulation Tissue Is Similar in Both Steel and Plasma Wound Beds on Days 8 and 20 Post-Incision, but Steel Incisions Have a Significantly Greater Number of Mast Cells

**Figure 5:**
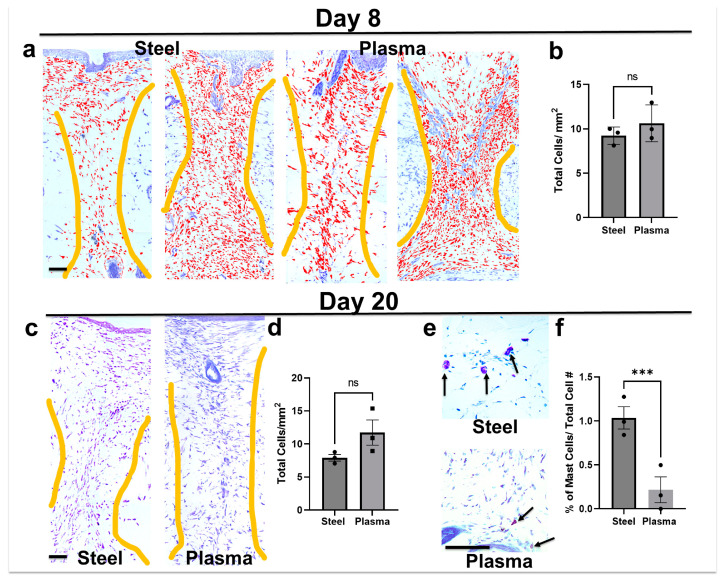
Comparison of cellular infiltration and mast cells in granulation tissue of steel or plasma scalpel wounds on days 8 and 20 post-incision. (**a**) Examples of more- and less-healed toluidine blue-stained granulation tissue from day 8 wound beds used to analyze cellular infiltrate by automated image analysis (red color indicates counted cells and yellow lines define granulation tissue area; mag. bar = 1 mm). (**b**) Graphed results indicate no significant (ns) differences (*p* = 0.054). (**c**) Comparisons of day 20 post-incision images used to analyze total cell count (mag. bar = 1 mm). (**d**) This analysis also resulted in no significant difference. (**e**) Toluidine blue images of larger pink/purple-stained mast cells observed on day 20 (arrows) (mag. bar = 1 mm). (**f**) Graphed results and paired *t*-tests indicate significantly more mast cells in the granulation tissue of steel scalpel wounds day 20 post-incision (***, *p*= 0.005). Average quantitation of 3-5 images for 3 separate rats (shown by black dot or squares) on each graph.

Toluidine blue is a stain for mast cells, which turn a dark or pink/purple color; their granular morphology is identifiable with light microscopy, while all nucleated cells stain light blue. Thus, this stain was used to quantitate total cellular infiltrate into the granulation tissue (demarcated by yellow drawn lines) on day 8 (Figure 5a,b) and day 20 (Figure 5c,d) post-incision. As previously mentioned, on day 8, there is a range of healing along each incision, and examples of more and less healing are shown for each scalpel type. Counted cells are pseudo-colored red to illustrate examples of automated counting with the image analysis macro. When the average number of cells in the granulation area were counted in images taken from at least 4 sections/rat (n = 3 rats) and a paired analysis was performed, no significant difference was observed (Figure 5b). A similar analysis performed on day 20 post-incision (Figure 5c; without the pseudo-coloring) also showed no significant difference (Figure 5d). Mast cells in toluidine blue-stained sections were larger and darker; when the same sections were analyzed using filters that isolated these cells, significantly more were observed in the granulation tissues from day 20 steel scalpel incisions (Figure 5e). A mean of 1.1 ± 0.31 mast cells/total cells were present when incisions were created with a steel scalpel (Figure 5f), while incisions made with the plasma scalpel had significantly less (0.25 ± 0.15 mast cells/total cells; *p* = 0.005).

## 4. Discussion

The purpose of this study was to determine the impact of cold plasma (J-Plasma) during surgical incision on the skin while evaluating wound healing and bactericidal activity when compared to the current standard of care of the surgical steel scalpel. We hypothesized that cold-activated plasma could be used to make full-thickness skin incisions and decrease bacterial burden within deep surgical sites without negatively impacting wound healing. This study found that full-thickness skin incisions by J-Plasma heal both externally and internally in a manner similar to wounds created by a steel blade scalpel with the added benefit of increased hemostasis.

A recent study by Pinelli et al. demonstrated optimal settings for deep dissection using the J-Plasma device for fibula free flap (J-Plasma at 4 L/min flow and 30% power), radial free forearm flap (J-Plasma at 3 L/min flow and 20% power), and latissimus dorsi flap (J-Plasma at 4 L/min and 50% power) harvests [7]. Our results, similar to their settings for human tissue dissection, suggest that 3 L/min flow and 40% power is the optimal choice for the maximum generation of nitrite and peroxide while also allowing for precise control with no visible tissue eschar.

Prior studies have demonstrated cold plasma to be effective in hemostasis [2,3,31,32]. While the standard steel scalpel must rely on the subject’s natural coagulation cascade and adequate surgical technique for hemostasis, cold plasma exhibits hemostatic qualities. Additionally, if required, bipolar electrocautery is routinely used in surgical settings to control hemostasis; however, as we have shown in our porcine model, this is much less conducive to efficient wound healing when creating the surgical incision through the dermis. The 2017 study by Bekeschus et al. hypothesized that plasma-derived ROS/RNS oxidize platelets, thereby increasing their activation, finally mediating hemostasis. The study found profound hemostatic activity utilizing a cold plasma device during liver resection in a murine model with a result comparable to electrocauterization. Our study demonstrates similar findings, with increased hemostasis utilizing J-Plasma to create full-thickness skin incisions compared to the standard steel scalpel in both an in vivo porcine model and in vivo rat model. Our study also demonstrates comparable hemostasis when comparing electrocautery during skin incision in an in vivo porcine model without the formation of visible necrotic tissue and better tissue healing (Figure 2). Utilizing a cold plasma scalpel could improve workflow efficiency during incision and lead to less blood loss while reducing the steps and the number of instruments used (i.e., superficial and deep scalpel blades, as well as a separate electrocautery device). The dissemination of pathogens such as C. acnes into deeper tissues layers during skin incision is a recognized clinical problem associated with increased surgical site infections and has been previously reported [33].

Numerous studies have shown that cold plasma (supplied by various plasma devices) may accelerate wound healing [34,35,36] and decrease bacterial inoculate of both natural wound flora and infected wounds [37,38]. In our study, J-Plasma incisions were found to have no difference in external wound appearance using previously published scoring systems [28,39] when compared to steel scalpel incisions at all time points throughout the study. Histologic analyses also found no difference at each time point with regards to granulation tissue, mature to immature collagen ratio, or collagen tissue width on day 8 across both treatment groups in the rat study.

A recent study by Wang et al. [40] observed the efficacy of non-thermal plasma, also known as cold plasma, on healing in an acute rat wound model. Two full-thickness dorsal cutaneous wounds of rats were treated with either a non-thermal helium plasma jet or helium. Their study found that compared to the control group, the wound area in the treatment group was significantly smaller on days 5, 7, and 14. The time for wound healing in the treatment group was also 2 days shorter compared to the control group. In addition, H&E staining of the scar sample on day 21 revealed that the scar width was not only smaller but also exhibited superior re-epithelialization compared to the control group. Our study varies from Wang et al. as the wounds were not treated multiple times with varying lengths of cold plasma application times but rather created by it. Our study does demonstrate, however, that compared to the standard of care, J-Plasma does not cause increased collagen formation or a change in the mature to immature collagen ratio and does not cause increased scar formation or delay wound healing compared to full-thickness incisions created by the steel scalpel.

Another study by Lee et al. [41] identified decreased numbers of mast cells and eosinophils along with lower epidermal thickness after the treatment of mice skin with non-thermal plasma (NTP). They determined that NTP suppresses mast cell activation, which is important for the allergic response, and ameliorates an atopic dermatitis-like skin inflammatory disease in mice. Our study agrees with these previous findings as scalpel incisions were found to have significantly higher counts of mast cells per millimeter than plasma incisions. High numbers of mast cells have also been observed in chronic wounds, hypertrophic scars, and keloids and have been implicated in fibrosis [42] and abnormal healing that could indicate scar tissue formation [43]. However, mast cells are also important for protecting against infection [44] and have been implicated in late-stage collagen remodeling, which fits with the time we observed the increased numbers.

Notable strengths of this study include the novel use of an FDA-approved device for the creation of full-thickness skin incisions, expanding its potential clinical utility. Our investigations employed a clinically relevant porcine model for select experiments of this study while resorting to a lower vertebrate model (rat) for the experiments that required an increased number of animals using standardized protocols regarding the collection and analysis of wounds, including their external appearance, collagen content, granulation tissue width, and H&E staining protocols.

## 5. Conclusions

The results of this study indicate surgical incisions created with cold plasma scalpel heal in a manner comparable to incisions created with a steel scalpel. In some instances, the creation of a surgical incision that exhibits enhanced hemostasis compared to the steel scalpel could be beneficial, specifically in areas where high levels of contamination from subdermal pathogens, such as the shoulder, groin, and spine, as the observed sealing of collagen fibrils and hemostasis may translate to a decreased rate of surgical site infection. Further studies are necessary to determine the clinical efficacy of cold plasma in human subjects.

## Figures and Tables

**Table 1:** Modified Southampton Scoring System.

Southampton Scoring System	
Grade	Appearance
0 Normal Healing	
(1) Normal Healing with mild bruising or erythema	0.25 some bruising
	0.50 considerable bruising
	0.75 mild erythema
(2) Erythema plus other signs of inflammation	0.25 at one point
	0.50 around sutures
	0.75 along wound
	0.95 around wound
(3) Clear or hemoserous discharge	0.25 at one point only (<2 cm)
	0.50 along wound (>2 cm)
	0.75 large volume
	0.95 prolonged (>3 days)
(4) Pus/purulent discharge	0.25 at one point only (<2 cm)
	0.50 along wound (>2 cm)
(5) Deep or severe wound infection with or without tissue breakdown	

## Data Availability

Data supporting this study is available through Jefferson University or the corresponding author.

## References

[1] Dandu N., Nelson B.B., Easley J.T., Huddleston H.P., DeFroda S.F., Bisazza K.T., Garrigues G.E., Yanke A.B. (2022). Quantifying the Magnitude of Local Tendon Injury from Electrosurgical Transection. J. Shoulder Elbow Surg..

[2] Bekeschus S., Brüggemeier J., Hackbarth C., Von Woedtke T., Partecke L.-I., Van Der Linde J. (2017). Platelets Are Key in Cold Physical Plasma-Facilitated Blood Coagulation in Mice. Clin. Plasma Med..

[3] Bekeschus S., Clemen R., Metelmann H.-R. (2018). Potentiating Anti-Tumor Immunity with Physical Plasma. Clin. Plasma Med..

[4] Braný D., Dvorská D., Halašová E., Škovierová H. (2020). Cold Atmospheric Plasma: A Powerful Tool for Modern Medicine. Int. J. Mol. Sci..

[5] Hertwig C., Meneses N., Mathys A. (2018). Cold Atmospheric Pressure Plasma and Low Energy Electron Beam as Alternative Nonthermal Decontamination Technologies for Dry Food Surfaces: A Review. Trends Food Sci. Technol..

[6] Taheri A., Mansoori P., Sandoval L.F., Feldman S.R., Pearce D., Williford P.M. (2014). Electrosurgery. J. Am. Acad. Dermatol..

[7] Pinelli M., Starnoni M., De Santis G. (2020). The Use of Cold Atmospheric Plasma Device in Flap Elevation. Plast. Reconstr. Surg.-Glob. Open.

[8] Filis K., Galyfos G., Sigala F., Zografos G. (2020). Utilization of Low-Temperature Helium Plasma (J-Plasma) for Dissection and Hemostasis during Carotid Endarterectomy. J. Vasc. Surg. Cases Innov. Tech..

[9] Gan L., Zhang S., Poorun D., Liu D., Lu X., He M., Duan X., Chen H. (2018). Medical Applications of Nonthermal Atmospheric Pressure Plasma in Dermatology. JDDG J. Dtsch. Dermatol. Ges..

[10] Musavi E.S., Khorashadizadeh S.M., Fallah R., Rahmanian Sharifabad A. (2019). Effect of Nonthermal Atmospheric Pressure Plasma on Plasma Coagulation in Healthy Persons and Patients under Treatment with Warfarin. Contrib. Plasma Phys..

[11] Nguyen L., Lu P., Boehm D., Bourke P., Gilmore B.F., Hickok N.J., Freeman T.A. (2018). Cold Atmospheric Plasma Is a Viable Solution for Treating Orthopedic Infection: A Review. Biol. Chem..

[12] Weltmann K.-D., Von Woedtke T. (2017). Plasma Medicine—Current State of Research and Medical Application. Plasma Phys. Control. Fusion.

[13] Bourke P., Ziuzina D., Han L., Cullen P.J., Gilmore B.F. (2017). Microbiological Interactions with Cold Plasma. J. Appl. Microbiol..

[14] Chernets N., Kurpad D.S., Alexeev V., Rodrigues D.B., Freeman T.A. (2015). Reaction Chemistry Generated by Nanosecond Pulsed Dielectric Barrier Discharge Treatment Is Responsible for the Tumor Eradication in the B16 Melanoma Mouse Model: DBD Generated ROS Eliminates Melanoma. Plasma Process. Polym..

[15] Gilmore B.F., Flynn P.B., O’Brien S., Hickok N., Freeman T., Bourke P. (2018). Cold Plasmas for Biofilm Control: Opportunities and Challenges. Trends Biotechnol..

[16] Eisenhauer P., Chernets N., Song Y., Dobrynin D., Pleshko N., Steinbeck M.J., Freeman T.A. (2016). Chemical Modification of Extracellular Matrix by Cold Atmospheric Plasma-Generated Reactive Species Affects Chondrogenesis and Bone Formation. J. Tissue Eng. Regen. Med..

[17] Graves D.B. (2017). Mechanisms of Plasma Medicine: Coupling Plasma Physics, Biochemistry, and Biology. IEEE Trans. Radiat. Plasma Med. Sci..

[18] Steinbeck M.J., Chernets N., Zhang J., Kurpad D.S., Fridman G., Fridman A., Freeman T.A. (2013). Skeletal Cell Differentiation Is Enhanced by Atmospheric Dielectric Barrier Discharge Plasma Treatment. PLoS ONE.

[19] Von Woedtke T., Weltmann K.-D. (2016). Grundlagen der Plasmamedizin. MKG-Chirurg.

[20] Chernets N., Zhang J., Steinbeck M.J., Kurpad D.S., Koyama E., Friedman G., Freeman T.A. (2015). Nonthermal Atmospheric Pressure Plasma Enhances Mouse Limb Bud Survival, Growth, and Elongation. Tissue Eng. Part A.

[21] Leutner S., Eckert A., Müller W.E. (2001). ROS Generation, Lipid Peroxidation and Antioxidant Enzyme Activities in the Aging Brain. J. Neural Transm..

[22] Bekeschus S., Brüggemeier J., Hackbarth C., Weltmann K.-D., Von Woedtke T., Partecke L.-I., Van Der Linde J. (2018). The Feed Gas Composition Determines the Degree of Physical Plasma-Induced Platelet Activation for Blood Coagulation. Plasma Sources Sci. Technol..

[23] Wende K., Bekeschus S., Schmidt A., Jatsch L., Hasse S., Weltmann K.D., Masur K., von Woedtke T. (2016). Risk Assessment of a Cold Argon Plasma Jet in Respect to Its Mutagenicity. Mutat. Res. Toxicol. Environ. Mutagen..

[24] Elston M.J., Dupaix J.P., Opanova M.I., Atkinson R.E. (2019). Cutibacterium Acnes (Formerly Proprionibacterium Acnes) and Shoulder Surgery. Hawaii J. Health Soc. Welf..

[25] Saltzman M.D., Nuber G.W., Gryzlo S.M., Marecek G.S., Koh J.L. (2009). Efficacy of Surgical Preparation Solutions in Shoulder Surgery. J. Bone Jt. Surg..

[26] Seaton M., Hocking A., Gibran N.S. (2015). Porcine Models of Cutaneous Wound Healing. ILAR J..

[27] Campwala I., Unsell K., Gupta S. (2019). A Comparative Analysis of Surgical Wound Infection Methods: Predictive Values of the CDC, ASEPSIS, and Southampton Scoring Systems in Evaluating Breast Reconstruction Surgical Site Infections. Plast. Surg..

[28] Fearmonti R., Bond J., Erdmann D., Levinson H. (2010). A Review of Scar Scales and Scar Measuring Devices. Eplasty.

[29] Sultana J., Molla M.R., Kamal M., Shahidullah M., Begum F., Bashar M.A. (1970). Histological Differences in Wound Healing in Maxillofacial Region in Patients with or without Risk Factors. Bangladesh J. Pathol..

[30] van de Vyver M., Boodhoo K., Frazier T., Hamel K., Kopcewicz M., Levi B., Maartens M., Machcinska S., Nunez J., Pagani C. (2021). Histology Scoring System for Murine Cutaneous Wounds. Stem Cells Dev..

[31] Heslin C., Boehm D., Milosavljevic V., Laycock M., Cullen P.J., Bourke P. (2014). Quantitative Assessment of Blood Coagulation by Cold Atmospheric Plasma. Plasma Med..

[32] Kramer A., Lindequist U., Weltmann K.-D., Wilke C., von Woedtke T. (2008). Plasma Medicine—Its Perspective for Wound Therapy. GMS Krankenhaushygiene Interdiszip..

[33] Falconer T.M., Baba M., Kruse L.M., Dorrestijn O., Donaldson M.J., Smith M.M., Figtree M.C., Hudson B.J., Cass B., Young A.A. (2016). Contamination of the Surgical Field with Propionibacterium Acnes in Primary Shoulder Arthroplasty. J. Bone Jt. Surg..

[34] Bekeschus S., Seebauer C., Wende K., Schmidt A. (2018). Physical Plasma and Leukocytes—Immune or Reactive?. Biol. Chem..

[35] Bekeschus S., Von Woedtke T., Emmert S., Schmidt A. (2021). Medical Gas Plasma-Stimulated Wound Healing: Evidence and Mechanisms. Redox Biol..

[36] Shahbazi Rad Z., Abbasi Davani F., Etaati G. (2018). Determination of Proper Treatment Time for in Vivo Blood Coagulation and Wound Healing Application by Non-Thermal Helium Plasma Jet. Australas. Phys. Eng. Sci. Med..

[37] Darmawati S., Nasruddin N., Putri G.S.A., Iswara A., Kurniasiwi P., Wahyuningtyas E.S., Nurani L.H., Hayati D.N., Ishijima T., Nakatani T. (2021). Accelerated Healing of Chronic Wounds under a Combinatorial Therapeutic Regimen Based on Cold Atmospheric Plasma Jet Using Contact and Noncontact Styles. Plasma Med..

[38] Klebes M., Ulrich C., Kluschke F., Patzelt A., Vandersee S., Richter H., Bob A., Von Hutten J., Krediet J.T., Kramer A. (2015). Combined Antibacterial Effects of Tissue-tolerable Plasma and a Modern Conventional Liquid Antiseptic on Chronic Wound Treatment. J. Biophotonics.

[39] Bailey I.S., Karran S.E., Toyn K., Brough P., Ranaboldo C., Karran S.J. (1992). Community Surveillance of Complications after Hernia Surgery. BMJ.

[40] Wang X.-F., Fang Q.-Q., Jia B., Hu Y.-Y., Wang Z.-C., Yan K., Yin S.-Y., Liu Z., Tan W.-Q. (2020). Potential Effect of Non-Thermal Plasma for the Inhibition of Scar Formation: A Preliminary Report. Sci. Rep..

[41] Lee M.-H., Lee Y.S., Kim H.J., Han C.H., Kang S.U., Kim C.-H. (2019). Non-Thermal Plasma Inhibits Mast Cell Activation and Ameliorates Allergic Skin Inflammatory Diseases in NC/Nga Mice. Sci. Rep..

[42] Noli C., Miolo A. (2001). The Mast Cell in Wound Healing. Vet. Dermatol..

[43] Urb M., Sheppard D.C. (2012). The Role of Mast Cells in the Defence against Pathogens. PLoS Pathog..

[44] Iba Y., Shibata A., Kato M., Masukawa T. (2004). Possible Involvement of Mast Cells in Collagen Remodeling in the Late Phase of Cutaneous Wound Healing in Mice. Int. Immunopharmacol..

